# Gender Differences in Suicide and Homicide Rates in Mexico City during 2019

**DOI:** 10.3390/ijerph19148840

**Published:** 2022-07-21

**Authors:** Humberto Nicolini, Juan Pablo Sánchez-de la Cruz, Rosa Giannina Castillo Avila, María Lilia López-Narvaéz, Thelma Beatríz González-Castro, Sophia Chávez-Manjarrez, José Eduardo Montes-de-Oca, Jaime Martínez Magaña, Carlos Alfonso Tovilla-Zárate, Alma Delia Genis Mendoza

**Affiliations:** 1 Laboratorio de Genómica de Enfermedades Psiquiátricas y Neurodegenerativas, Instituto Nacional de Medicina Genómica, Ciudad de México 14610, Mexico; hnicolini@inmegen.gob.mx (H.N.); dramarilit@hotmail.com (S.C.-M.); ansesaos7@gmail.com (J.E.M.-d.-O.); jimy.10.06@gmail.com (J.M.M.); 2 División Académica Multidisciplinaria de Comalcalco, Universidad Juárez Autónoma de Tabasco, Comalcalco 86658, Mexico; jpsanchezc94@gmail.com; 3 División Académica de Ciencias de la Salud, Universidad Juárez Autónoma de Tabasco, Villahermosa 86100, Mexico; gianninaavila2012@hotmail.com; 4 Hospital Chiapas Nos Une Dr. Gilberto Gómez Maza, Secretaría de Salud de Chiapas, Tuxtla Gutiérrez 29045, Mexico; dralilialonar@yahoo.com.mx; 5 División Académica Multidisciplinaria de Jalpa de Méndez, Universidad Juárez Autónoma de Tabasco, Jalpa de Mendez 86205, Mexico; thelma.glez.castro@gmail.com; 6 Servicios de Atención Psiquiátrica, Hospital Psiquiátrico Infantil “Juan N. Navarro”, Mexico City 14080, Mexico

**Keywords:** suicide, homicide, México City, rates and gender

## Abstract

Suicides and homicides are public health problems around the world. The rates of suicide and homicide have increased in the past years. The objectives of this study are to estimate the rates of suicide and homicide in Mexico City, and to determine the rates of suicide and homicide by sex in the different municipalities of Mexico City during 2019. Data analyzed were obtained from files of governmental organizations in Mexico City. From the general victims-in-research-folders, we choose “victims of crime” or “loss of life by suicide” that happened in 2019. Sex and municipality of residence were obtained. The rate of suicide was of 5.65 cases per 100,000 habitants. Stratified by gender and by municipalities, the highest rates observed were 6.8 suicides per 100,000 males and 29.6 suicides per 100,000 females. The sex ratio was 4.2:1 (males: females). Regarding homicides, the rates were 16.68 homicides per 100,000 females and 67.41 homicides per 100,000 males. The Venustiano Carranza Municipality showed the highest homicide rate in men with 131.72 homicides per 100,000 males. The homicide sex ratio was 7.8:1. The findings of the present study highlight that death by suicide is more common in men with 7.8 more times than in women. The municipalities with higher deaths by suicide have lower rates of homicides and the municipalities with higher deaths by suicide showed lower rates of suicide in men.

## 1. Introduction

Violent deaths represent one of the main causes of death during the economically productive age [1]. In men, homicides and suicides represent one of the ten leading causes of death [1,2]. In this regard, homicide is defined as the unlawful death inflicted on a person with the intent to cause death or serious injury [3].

There are different types of homicides: (a) Intentional homicide occurs when total responsibility is focused on the aggressor [4]; (b) unintentional homicide can be categorized into negligent (when killing as a result of recklessness or negligence, for example driving while intoxicated and hitting a person) [5,6] and non-negligent, which occurs in response to mitigating circumstances such as provocation or diminished responsibility, for example armed conflicts or self-defense [6].

Criminal activity is the cause of many deaths, with an estimated global intentional homicide rate of approximately 5 deaths per 100,000 individuals [7]. Homicides are more frequent in low- and middle-income countries and less frequent in high-income countries. For instance, in the Latin American and Caribbean region, rates of 22 homicides per 100,000 inhabitants are reported. While in the European Union the lowest rate reported is 1 homicide per 100,000 inhabitants [7]. In Europe and Asia homicide rates are declining; however, in regions of the Americas and Africa, homicide rates continue to rise [8].

In Mexico, as in many Latin American countries, homicide rates have increased over the past decade [9] and nowadays are approximately five times higher than the world average. Although intentional homicide is a global issue, Central America (including Mexico) has the highest homicide rates in the world [10]. In addition, femicides (the most extreme form of violence against women) are prevalent in the Mexican population [11] some issues such as violence against women have been normalized in all areas of society [12]. Similarly, since a decade ago, the increase in the rate of homicidal violence has been studied, particularly in the young population, because it could be preventing the increase in life expectancy among Mexican men [13]. Therefore, it is important to study these issues that have directly affected the Mexican population for several years.

In this sense, in 2018, the homicide rate in Mexico was 29 per 100,000 inhabitants [9]. Current reports indicate that at least eight cities in Mexico are among the ten most violent in the world [14], with a rate that ranges from 100 to 195 per 100,000 inhabitants. In Mexico City, in 2019 the highest figure was reported with 1504 intentional homicides [14].

On the other hand, suicide is defined as the act of ending one’s own life [15] and it has been described that biological, environmental, and psychosocial factors intervene in suicidal behavior [15]. Additionally, suicide represents one of the three leading causes of death in the young adult population [16].

Suicide rates vary globally; nonetheless, they are higher in developed countries, high-income countries than in developing countries [16]. For example, in Eastern Europe, Southeast Asia, and countries such as South Korea and Russia, suicide rates exceed 20 cases per 100,000 inhabitants. While in North Africa, the Middle East and some Latin American countries, lower rates are reported with 5 cases per 100,000 inhabitants [17]. In Mexico for instance, the suicide rate is 6.2 per 100,000 inhabitants.

Suicide rates are higher in men than in women, with 10.4 suicides per 1,000,000 males and 2.2 suicides per 1,000,000 females [18]. The most affected age group is 18 to 29 years for both sexes. [19]. In this regard, in Mexico City an increase in the suicide rate of approximately 50% was observed since 2017 and this change is of particular interest to study.

On the other hand, homicides and suicides have been commonly studied as individual phenomena. However, there are a few reports that integrate both phenomena as a manifestation of violence. In this regard, factors that intervene in the development of both phenomena have not been fully elucidated; hence it is important to study the relationship between homicides and suicides [20].

It has been described that some social and cultural factors determine violent behavior that can be directed toward another person or toward oneself [20,21]. However, there are fewer studies that evaluate the rates of suicide and homicide in the Mexican population. It is interesting to know variations between the suicide and homicide rates, mainly in places where increases have been observed, such as in Mexico City. We hypothesize that suicide and homicide rates in Mexico City have increased in parallel in both men and women. Therefore, the objective of this study was to describe the differences between the homicide and suicide rates by gender in the different municipalities of Mexico City during 2019.

## 2. Materials and Methods

### 2.1. Study Design

This is an observational, retrospective study. We used figures gathered by governmental agencies in Mexico City, through an online search.

### 2.2. Participants

The participants of this study were selected from the open data of Victims in the Investigation Folder of the Attorney General Office of Mexico City that appear in the following link: https://datos.cdmx.gob.mx/dataset/victimas-en-carpetas-de-investigacion-fgj (accessed on 31 March 2022).

The last search to select the participants was performed on 31 December 2019 by two researchers (EMOJ and JPSC) [22].

### 2.3. Data Collection

Data on gender, deaths by suicide and intentional homicides were obtained from the “Victims of crime” database (CSV 138741896KB) [22]. The database was filtered, sectioning the information according to the crimes of interest. The information was then categorized according to sex. In the homicide variable, the legal status of “corpse” was selected. Likewise, in the suicide variable, “loss of life by suicide” was selected. The information obtained from the database was corroborated by two researchers (CATZ and ADGM).

Files without information regarding the sex of the victims, unintentional homicides due to a traffic accident or self-defense, and intentional homicides outside of Mexico City were excluded.

### 2.4. Statistical Analysis

Suicide rates and frequency of crimes were calculated based on the 2015 Intercensus Survey carried out by the National Institute of Statistics and Geography (INEGI). This survey is the last statistical update of the population in Mexico for the research period 2016–2018. The suicide–homicide relationship was calculated according to sex. The suicide–homicide relationship was calculated considering 100,000 Mexican individuals and the results were plotted using ggplot (data visualization package) of the statistical programming language R 4.0.2.

## 3. Results

### 3.1. Suicide Rates by Municipality

The suicide rate calculated for Mexico City was 6.07 cases per 100,000 inhabitants in 2019. The results obtained on suicide rates in Mexico City varied among municipalities. In this regard, the highest incidence of suicides was observed in Milpa Alta Municipality with a rate of 15.95 cases per 100,000 inhabitants. While the lowest incidence of suicides was observed in Tlalpan and Tláhuac Municipalities with 8.8 and 9.0 per 100,000 inhabitants, respectively. The suicide incidence in all municipalities was above the national average, which is 6.2 per 100,000 inhabitants.

### 3.2. Homicide Rates by Municipality

**Figure 1:**
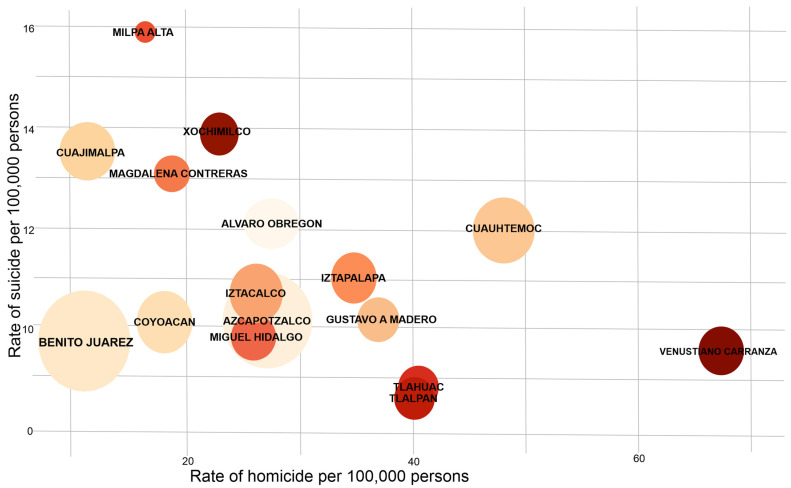
Comparison of homicide and suicide rates (per 100,000 inhabitants) in the general population of Mexico City in 2019, distributed by municipalities. The color is indistinct.

The rate of homicides calculated for Mexico City was 17.11 homicides per 100,000 inhabitants in 2019; this rate also varied among municipalities. The results showed ranges from 16.68 to 67.41 per 100,000 inhabitants. Figure 1 shows the distribution of suicide/homicide in the different municipalities of Mexico City. As an interesting fact, we observed that Milpa Alta Municipality showed the lowest homicide rate and, at the same time, the highest suicide rate. Similarly, Venustiano Carranza Municipality had the lowest suicide rate, but the highest homicide rate. 

### 3.3. Suicide and Homicide in Women by Municipality

**Figure 2:**
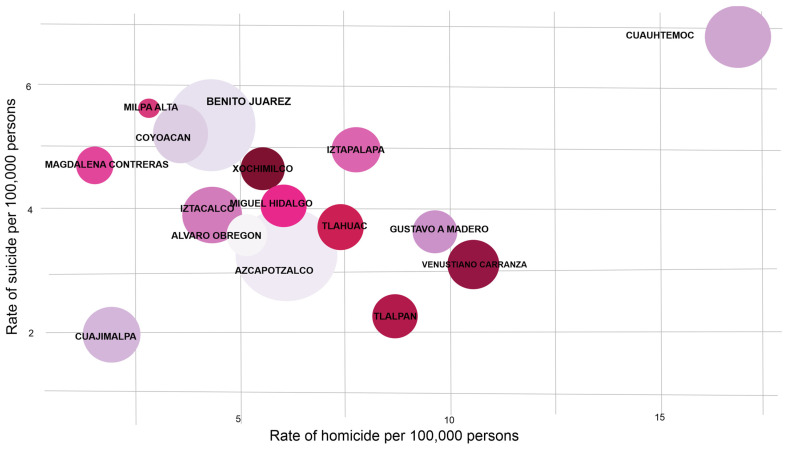
Suicide and homicide rates in women during the 2019.

Figure 2 shows the suicide/homicide rate in women in Mexico City. We observed that in 2019, the homicide rate for women in Mexico City was 2.5 per 100,000 females. In addition, the highest homicide rate (16.83 per 100,000 females) was observed in Cuauhtémoc Municipality. The suicide rate in women in Mexico City was 2.38 per 100,000 females and the highest suicide rate in women was also observed in Cuauhtémoc Municipality (6.8 per 100,000 females). For its part, in Cuajimalpa Municipality we observed the lowest rates of suicide and homicide with 1.93 per 100,000 females.

### 3.4. Suicide and Homicide in Men by Municipality

**Figure 3:**
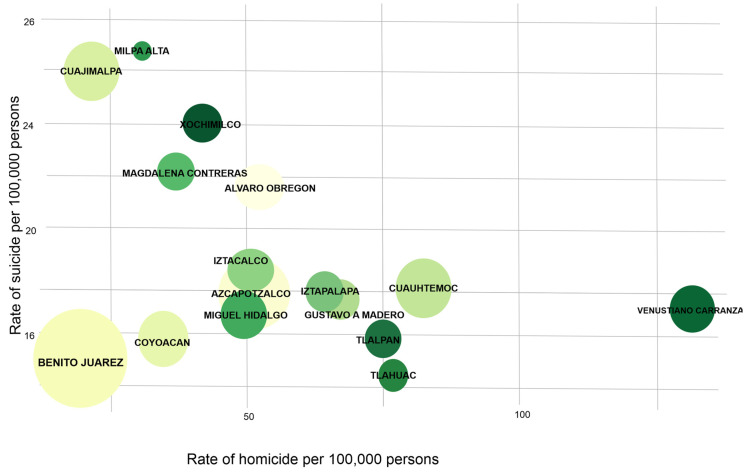
Suicide and homicide rates in men during the 2019.

The homicide rate for men in Mexico City was 32.96 per 100,000 males in 2019. In this sense, Venustiano Carranza Municipality showed the highest homicide rate in men (131.72 per 100,000 males), but it had the lowest suicide rate with 9.92 per 100,000 males. Whereas in Milpa Alta Municipality the highest suicide rate was 29.6 suicides per 100,000 males. Therefore, the homicide ratio by sex is 7.8:1 in men compared to women, and the suicide ratio by sex was 4.2 men for every woman Figure 3.

### 3.5. Suicide and Homicide Rates from 2016 to 2020

The Table 1 shows the suicide/homicide rate in Mexico City by gender in the period from 2016 to 2020. We observe a constant increase in homicide rates for the general population in Mexico City. In 2016, the rate was 11.49 and for the year 2020 it increased to 18.44. In one year, the homicide rate in women increased from 2.50 to 4.48, almost doubled. In five years, suicide rates in Mexico City also showed variations. The rate increased from 4.36 in 2016 to 6.13 in 2020. For years before 2019, homicide and suicide rates by age and sex could not be calculated because this information is not available in the databases.

## 4. Discussion

The objective of the present study was to describe the differences by gender in homicide and suicide rates per municipality in Mexico City. To our knowledge, this is the first study that analyzes this information.

Studies show that in Mexico, the homicide rate has increased over the years. A study performed between 1990 and 2009 showed a significant variation from 7.6 to 16.6 per 100,000 inhabitants [23]. Currently, the rate exceeds 26 per 100,000 inhabitants. Reports indicate that in the metropolitan area of Mexico City, the homicide rate increased to 13.28 homicides per 100,000 inhabitants in 2008 [24]. 

In this study, we observed that the homicide rate was 17.11 per 100,000 inhabitants in 2019, which represents a significantly higher figure compared to previous years. 

It was reported that the homicide rate in men increased in Mexico City from 14.2 per 100,000 males in 2005 to 16.2 homicides per 100,000 males in 2019 [24,25]; nonetheless, 16.2 is much lower than what we observed in our study, where the homicide rate was of 32.96 homicides per 100,000 males.

Fuentes and Sánchez et al., reported that in 2010 there were a total of 808 homicides in Mexico City and the municipalities with the highest number of homicides were Iztapalapa, Gustavo A. Madero, Cuauhtémoc, Benito Juárez, and Coyoacán with a repetition of the criminal pattern the following three years [25].

In our study, we identified that the municipalities with the highest homicide rates were: Venustiano Carranza, Cuauhtémoc, and Tláhuac. It is possible that there has been a migration phenomenon in the areas with the highest crime rate in recent years. In this sense, the causes of the increase in homicide rates are not clear, but they could be related to crime, unemployment, and social inequality.

We also observed an increase in suicide rates. It was estimated that from 2000 to 2012 the suicide rate increased approximately 17.1% [26]. In 2017, the suicide rate in Mexico was 5.31 per 100,000 inhabitants [27]. Furthermore, suicidal behavior incidence in Mexico City has also increased [26].

Historically, suicide rates in Mexico City are lower than the national average rate. We observed 5.65 suicides per 100,000 inhabitants in 2019, while in 2015, the suicide rate was 4.1 per 100,000 inhabitants [28]. The aforementioned shows a clear increase in the suicide rate in Mexico City. In 2015, the rate was 6.8 per 100,000 males and 1.7 per 100,000 females [28]. In 2019, we observed a rate of 9.92 per 100,000 males and 2.38 suicides per 100,000 females. A previous study identified that the municipalities with the highest suicide rates in men were: Cuauhtémoc (13.82), Magdalena Contreras (11.67), and Milpa Alta (10.47) (24); while for women, they were Miguel Hidalgo (3.43), Tlalpan (3.16), and Iztapalapa (2.52) [28].

Regarding differences by gender in 2019, we observed the highest suicide rate in men in Milpa Alta Municipality; while in women, the highest suicide rate was in Cuauhtémoc Municipality. These results show variability between suicide rates according to the municipalities in Mexico City as well as gender.

Currently, suicide represents one of the five main causes of death in the young population, both in men and women [29]. In Mexico City, deaths by suicide or by homicides are becoming more frequent. In this regard, violent deaths considerably increase the years of life lost due to premature death in both, women [30] and men, also affecting children and young adults [31]. 

Suicides and homicides are considered cases of violent death and studying suicide-homicide together is difficult. Several studies have described that both complex phenomena result from different situations and different settings [32,33]. It is possible that these acts arise in the personal setting (a person commits suicide); in the family sphere (marital homicide, domestic violence); and in the social sphere (shooting by firearm at work, school) [32]. The various forms of violence broaden the range of victims, such as in the cases of multiple homicides, terrorist attacks and wars.

In Mexico, although the behaviors above described are rare in comparison to the multi-homicides in the United States of America or to Guerrillas conflicts in Colombia; the suicide–homicide phenomena continue to increase.

One proposal to prevent deaths by homicide and suicide is the implementation of rigorous laws to control the use and possession of firearms in the general population, in addition to providing a psychosocial evaluation and training to those who are granted a license to own and/or carry weapons. However, the consequences of implementing these measures vary considerably among different populations. For example, in Canada, this measure failed to reduce suicide and homicide rates [34]. In Austria, due to internal and European Union regulations on firearm possession, less availability was associated with a decrease in suicides and homicides [35,36].

In Australia, changes in mass shooting incidents, in firearm death rates, suicides, and homicides were observed after changes in the firearms regulation were implemented by the Australian Government [37]. Therefore, we believe that increasing police surveillance and having stronger laws against people carrying firearms should be implemented in Mexico City.

Some study limitations should be taken into consideration: (a) It was not possible to perform an analysis by age group; (b) there were unidentified cases of homicide and no records of age in the database; for this reason, it was not possible to determine the age group to which they belonged and as a consequence, the sample decreased; (c) the scope in time can be considered short (2019); however, our study provides a more up to date report; (d) due to the characteristics of the study, it was not possible to evaluate sociodemographic variables; (e) it was not possible to perform other evaluations such as the psychological autopsy to elucidate factors that intervene in the increased rates of both phenomena.

Our study also has strengths: (a) This study is one of the few ones that analyzes the relationship between homicide and suicide; (b) this study shows in detail the differences between homicide/suicide in Mexico City by municipalities and gender; (c) this study analyzed the trend of homicide and suicide rates in Mexico City; (d) our results generate an initial statistical contribution of the homicide/suicide relationship in the Mexico City population.

## 5. Conclusions

In conclusion, our data indicates differences by gender and by municipalities in suicides and homicides. Deaths by suicide were higher in men than in women, with a sex-ratio of 4.2:1. Whereas the homicide rate was 7.8 more times in man than that in women. The homicide rate in women increased more than that of men; however, the homicide rate for men is considerably higher. The municipalities with higher deaths by suicide also had lower rates of homicides, and municipalities with higher deaths by suicide showed lower rates of suicide. More studies that measure and evaluate factors associated with these rates are necessary.

## Figures and Tables

**Table 1:** Homicide and suicide rates in Mexico City by gender in the period from 2016 to 2020.

Calculated Rate *		Years Analyzed				
		2016	2017	2018	2019	2020
Homicide	General population	11.49	13.63	15.94	17.11	18.44
	Male	NA	NA	NA	32.96	33.34
	Female	NA	NA	NA	2.50	4.48
Suicide	General population	4.36	4.21	4.41	6.07	6.13
	Male	NA	NA	NA	9.92	10.2
	Female	NA	NA	NA	2.38	2.43

* Rate calculated per 100,000 inhabitants. NA: no data available.

## Data Availability

Not applicable.
